# Mutations in viral nucleocapsid protein and endoRNase are discovered to associate with COVID19 hospitalization risk

**DOI:** 10.1038/s41598-021-04376-4

**Published:** 2022-01-24

**Authors:** Lue Ping Zhao, Pavitra Roychoudhury, Peter Gilbert, Joshua Schiffer, Terry P. Lybrand, Thomas H. Payne, April Randhawa, Sara Thiebaud, Margaret Mills, Alex Greninger, Chul-Woo Pyo, Ruihan Wang, Renyu Li, Alexander Thomas, Brandon Norris, Wyatt C. Nelson, Keith R. Jerome, Daniel E. Geraghty

**Affiliations:** 1 Division of Public Health Sciences, Fred Hutch Cancer Center, Seattle, WA 98109 USA; 2 Vaccine and Infectious Disease Division, Fred Hutch Cancer Center, Seattle, WA 98109 USA; 3 Department of Laboratory Medicine and Pathology, University of Washington School of Medicine, Seattle, WA USA; 4 Clinical Research Division, Fred Hutch Cancer Center, Seattle, WA 98109 USA; 5 Quintepa Computing LLC, Nashville, TN USA; 6 Department of Chemistry, Department of Pharmacology, Vanderbilt University, Nashville, TN USA; 7 Department of Medicine, University of Washington School of Medicine, Seattle, WA USA; 8 Scisco Genetics Inc., Seattle, WA 98102 USA

**Keywords:** Computational biology and bioinformatics, Genetics, Molecular biology, Diseases

## Abstract

SARS-CoV-2 is spreading worldwide with continuously evolving variants, some of which occur in the Spike protein and appear to increase viral transmissibility. However, variants that cause severe COVID-19 or lead to other breakthroughs have not been well characterized. To discover such viral variants, we assembled a cohort of 683 COVID-19 patients; 388 inpatients (“cases”) and 295 outpatients (“controls”) from April to August 2020 using electronically captured COVID test request forms and sequenced their viral genomes. To improve the analytical power, we accessed 7137 viral sequences in Washington State to filter out viral single nucleotide variants (SNVs) that did not have significant expansions over the collection period. Applying this filter led to the identification of 53 SNVs that were statistically significant, of which 13 SNVs each had 3 or more variant copies in the discovery cohort. Correlating these selected SNVs with case/control status, eight SNVs were found to significantly associate with inpatient status (q-values < 0.01). Using temporal synchrony, we identified a four SNV-haplotype (t19839-g28881-g28882-g28883) that was significantly associated with case/control status (Fisher’s exact *p* = 2.84 × 10^–11^). This haplotype appeared in April 2020, peaked in June, and persisted into January 2021. The association was replicated (OR = 5.46, *p*-value = 4.71 × 10^−12^) in an independent cohort of 964 COVID-19 patients (June 1, 2020 to March 31, 2021). The haplotype included a synonymous change N73N in endoRNase, and three non-synonymous changes coding residues R203K, R203S and G204R in the nucleocapsid protein. This discovery points to the potential functional role of the nucleocapsid protein in triggering “cytokine storms” and severe COVID-19 that led to hospitalization. The study further emphasizes a need for tracking and analyzing viral sequences in correlations with clinical status.

## Introduction

Severe acute respiratory syndrome coronavirus 2 (SARS-CoV-2), initially reported in Wuhan, Hubei, People’s Republic of China^1^, is the causal pathogen for the coronavirus disease (COVID-19), causing over 5 million fatalities worldwide as of November 2021 (covid19.who.int). In the United States, COVID-19 has infected more than 47 million people and claimed over 750,000 lives as of this date (covid.cdc.gov). In Washington State, where the first COVID-19 patient in the US was reported on January 19, 2020, at least 9100 patients have died, among 670,000 confirmed infections (www.doh.wa.gov/Emergencies/COVID19). Like other viruses, SARS-CoV-2 accumulates mutations with each cycle of replication known as single nucleotide variants (SNVs). Based on mutational frequencies or associated phenotypes, a viral strain with one or more such SNVs are referred to as variants, and variants meeting specific criteria can be classified as either Variants of Interest (VOI), Variants of Concern (VOC) or Variants of High Consequence (VOHC) by the Centers for Disease Control and Prevention (CDC) (https://www.cdc.gov/coronavirus/2019-ncov/cases-updates/variant-surveillance/variant-info.html). Currently, three lineages are classified as VOI (B.1.526-lota, B.1.525-Eta and P.2-Zeta), and five lineages as VOC (B.1.1.7-Alpha, P.1-Gamma, B.1.351-Beta, B.1.427-Epsilon, B.1.429-Epsilon, and, recently, B.1.617-Kappa/Delta), because of their elevated transmissibility or impact on neutralization^2,3^. The delta variant (B.1.617.2) was initially reported in India and was found to spread throughout the world with substantial transmissibility (https://www.cdc.gov/coronavirus/2019-ncov/variants/variant.html). At this point, no VOHC has been declared by CDC.

The timely identification of VOHC is essential for the public health response against COVID-19 and requires viral genomic research directly connected to appropriately acquired clinical phenotypes in the clinical setting. Here our primary interest was to discover viral SNVs that associate with the COVID-19 hospitalization risk. We report results identifying SNVs that associated with COVID-19 hospitalization risk, through a discovery case–control study of COVID-19 patients whose viral genomes were sequenced and whose hospitalization status (inpatient versus outpatient) and demographic data were retrieved from a database of COVID testing request forms. To improve analytical power, we utilized a large collection of viral sequences from 7137 Washington residents deposited in GISAID and selected those SNVs that showed significant and substantial expansions from January 2020 to 2021. By correlating these identified SNVs with hospitalization status in the discovery study, we identified those SNVs that associated with hospitalization risk. The identified SNPs were subsequently replicated in an independent cohort.

## Results

### SNVs with significant and substantial expansions

**Figure 1:**
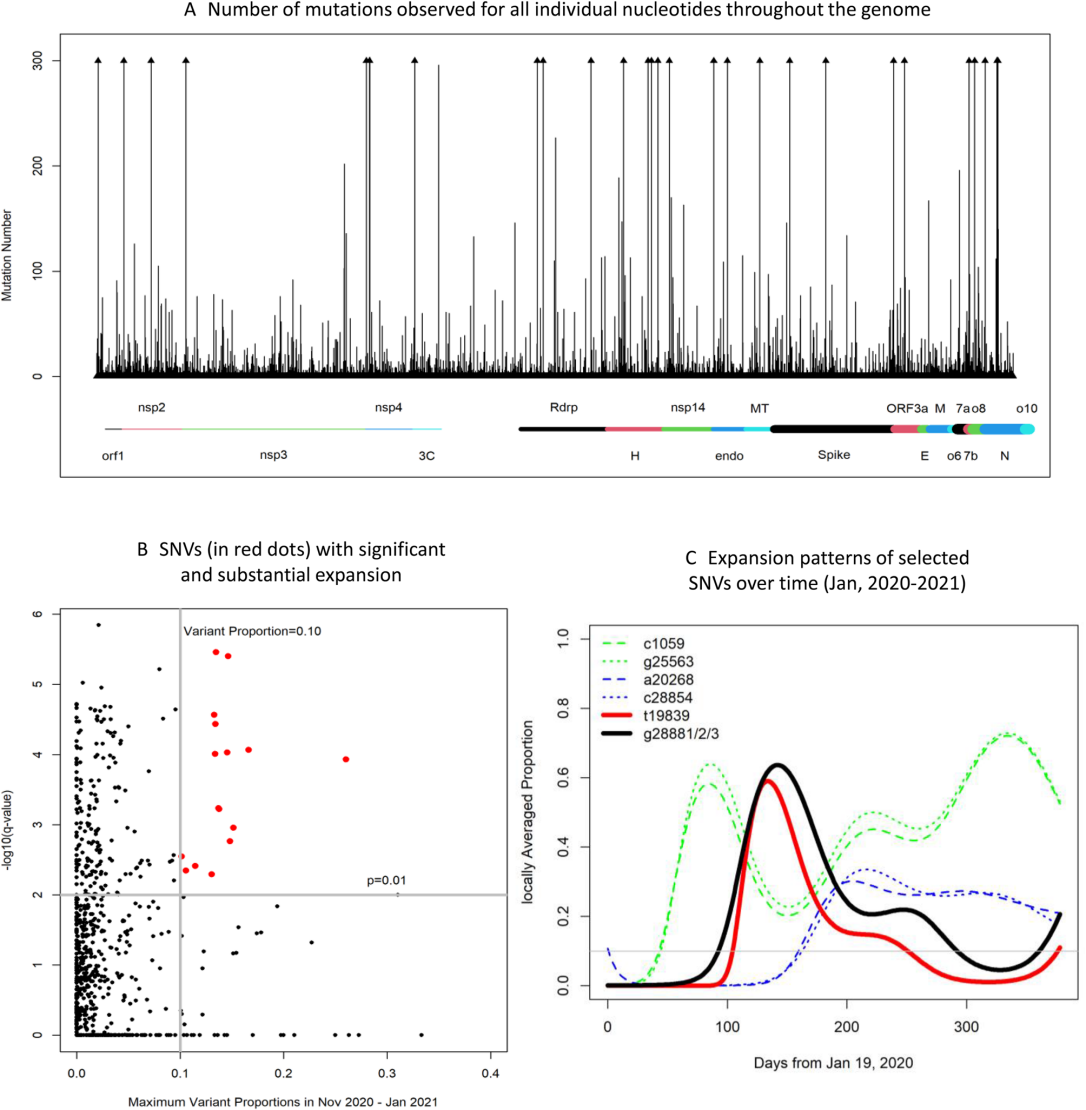
Results from analyzing 7137 viral genomes sequenced by laboratories in Washington state and deposited to GISAID. (**A**) Results from counting mutational numbers per nucleotide throughout the viral genome. Upper arrow indicates observed counts greater than 300. The viral genome is annotated with gene designations immediately below. (**B**) Computed q-values and maximum values of variant proportions in November 2020, December 2020, and January 2021, obtained from fitting generalized linear models to all individual SNVs. SNVs exceeding established threshold q-value and maximum proportions are highlighted in red (upper right corner). (**C**) Eight selected SNVs with significant and substantial temporalities are mapped using their locally averaged variant proportions over time from fitted generalized linear models (color key upper left).

The SARS-COV-2 accumulates mutations during replication, potentially generating new strains, some of which have undergone substantial expansions in the population either because of super-spreader events or due to functional changes resulting in elevated transmissibility. Here we considered an SNV to be of interest if it had a statistically significant expansion in the study period and its average proportion of mutations in the last three months of the study period exceeded 10%. To identify such SNVs, we utilized 7137 viral genomes that had been generated from laboratories in Washington state and deposited to GISAID and that had been aligned and subjected to quality control (see “Materials and methods”). Comparing all viral genome sequences to the reference sequence led to counts of mutations at each nucleotide in the genome shown in Fig. 1A. This analysis showed that the Spike protein had two common SNVs with 300 or more copies and the nucleocapsid (N) protein had multiple common SNVs. Since over 90% of positions in the 30 Kb genome had fewer than 3 mutations, we focused on the remaining ~ 10% (2516 nucleotides) for possible significant expansions. A non-linear logistic regression model to regress the binary indicator for mutation at each selected nucleotide on the collection time was then employed. In essence, this model fitted locally averaged proportions of mutants throughout the study period. If an SNV had undergone expansion, its locally averaged proportion would increase over time, as the mutant type became more common in the population.

This temporal expansion, deviating from random fluctuation, was quantified by a non-parametrically estimated function and its statistical significance was quantified by the *p*-value. To account for multiple comparisons, *p*-values were then converted to false positive error rates, i.e., q-values. An SNV was deemed to have a significant expansion if the q-value was less than 0.01. Meanwhile, to focus on those pertinent SNVs that emerged or maintained their dominance in the population, fitted models were used to compute locally averaged proportions daily and calculate the maximum proportion in last three months, denoted as Pmax. An SNV was deemed to have substantial expansion if the Pmax exceeded 10%. Figure 1B shows q-values and Pmax from 2516 nucleotides, among which 53 nucleotides met the established threshold values (q-value < 0.01 and Pmax > 0.10, supplementary Table S1). Notably, four coding SNVs and 1 non-coding SNV were identified in the Spike protein, and SNVs with substantial expansions occurred in other ORFs/genes as well.

### Variants in nucleocapsid, endoRNase and ORF3a have significant associations with COVID-19 hospitalization risk

The central goal of this study was to correlate identified SNVs with the hospitalization risk in the discovery case–control samples in Washington state. The discovery cohort included 295 outpatients (controls) and 388 inpatients (cases) for whom SARS-CoV-2 genome sequences were obtained (Table 1). Given constraints on the assembly of nasal swab samples and extraction of clinical data from the operational database, the case–control study took available samples from COVID-19 patients, while attempting to balance cases and controls for sex, age and collection times. Nevertheless, there were some imbalances in collection times for outpatients from March to June and for inpatients from March to August.

**Table 1:** Descriptive statistics of 683 and 964 participating patients in, respectively, the discovery and replication case–control studies.

Variable	Description	Discovery (n = 295 + 388)			Replication (n = 476 + 488)		
		OUT	IN	*p*-value	OUT	IN	*p*-value
Sex	Female	170 (57.63)	184 (47.42)	1.69E−02	216 (45.38)	231 (47.34)	2.39E−26
	Male	124 (42.03)	200 (51.55)		177 (37.18)	256 (52.46)	
	UNK	1 (0.34)	4 (1.03)		83 (17.44)	1 (0.20)	
Age (year)	1-	9 (3.06)	29 (7.47)	9.53E−04	112 (23.53)	73 (14.96)	1.15E−06
	20-	112 (38.1)	132 (34.02)		213 (44.75)	180 (36.89)	
	40-	139 (47.28)	151 (38.92)		105 (22.06)	148 (30.33)	
	60–100	34 (11.56)	76 (19.59)		45 (9.45)	86 (17.62)	
	UNK	1 (0.34)			1 (0.21)	1 (0.20)	
Collection	March	167 (56.61)	178 (45.88)	5.00E−04			
Month	April	113 (38.31)	72 (18.56)				
	May	12 (4.07)	24 (6.19)				
	June	3 (1.02)	14 (3.61)		179 (37.61)	111 (22.75)	5.00E−06
	July		96 (24.74)		24 (5.04)	29 (5.94)	
	August		4 (1.03)		104 (21.85)	137 (28.07)	
	September				21 (4.41)	16 (3.28)	
	October				27 (5.67)	14 (2.87)	
	November				57 (11.97)	6 (1.23)	
	December				4 (0.84)	10 (2.05)	
	January				12 (2.52)	109 (22.34)	
	February				48 (10.08)	56 (11.48)	

To ensure the robustness of the association analysis on individual SNVs, we limited our analysis to 13 SNVs that had ten or more mutants observed (Table 2). Correlating these SNVs with case/control status through an unadjusted logistic regression model, we estimated coefficient (log odds ratio), standard error, Z value, *p*-value and q-value. For two SNVs with zero occurrences among outpatients, we performed the Fisher’s exact test, where the logistic regression was not appropriate. Results from the adjusted logistic regression analysis are presented in the middle of Table 2. For readability, we highlighted q-values that were less than 0.01 using bold to correspond to positive and negative Z-values, reflecting the increase or decrease of COVID-19 hospitalization risk.

**Table 2:** Association results of selected SNVs (with at least 10 mutations) with hospitalization status (inpatient vs outpatient) in a case–control study of 683 Covid 19 cases: frequencies of wildtypes/mutations among outpatients and inaptients, estimated coefficients, standard errors, *p*-values, q-values, corresponding residue (if SNV is in the coding region), indicators for urgent SNVs, and corresponding genes.

ID	SNV	Wild/mutant		Unadjusted analysis					Adjusted analysis*					Residue	Genes
		OUT	IN	Coef	SE	Z	*p*	q	Coef	SE	Z	*p*	q		
1	c241	88/207	101/287	0.19	0.17	1.10	2.72E−01	3.93E−01	0.22	0.17	1.28	1.99E−01	2.93E−01		
2	c1059	102/193	207/181	− 0.77	0.16	− 4.85	1.24E−06	**8.06E−06**	− 0.79	0.16	− 4.94	7.98E−07	**4.15E−06**	T85I	nsp2
3	c3037	86/209	101/287	0.16	0.17	0.91	3.65E−01	4.74E−01	0.20	0.18	1.12	2.62E−01	3.41E−01	F106F	nsp2
4	c14408	85/210	101/287	0.14	0.17	0.81	4.19E−01	4.95E−01	0.18	0.18	1.00	3.19E−01	3.77E−01		
5	t19839	295/0	345/43	> > 0			**1.09E−11**		> > 0					N73N	endoRNase
6	a20268	294/1	343/45	3.65	1.01	3.60	3.16E−04	**6.85E−04**	3.76	1.02	3.70	2.15E−04	**4.67E−04**	L216L	endoRNase
7	a23403	88/207	101/287	0.19	0.17	1.10	2.72E−01	3.93E−01	0.22	0.17	1.27	2.03E−01	2.93E−01	D614G	S-spike-protein
8	g25563	98/197	204/184	− 0.80	0.16	− 5.01	5.52E−07	**7.18E−06**	− 0.83	0.16	− 5.11	3.24E−07	**4.15E−06**	Q57H	ORF3a
9	c27964	288/7	370/18	0.69	0.45	1.53	1.25E−01	2.32E−01	0.70	0.45	1.55	1.22E−01	2.26E−01	S24L	ORF8
10	c28854	295/0	346/42	> > 0			**2.03E−11**		> > 0					S194L	Nucleocapsid
11	g28881	287/8	334/54	1.76	0.39	4.54	5.66E−06	**1.47E−05**	1.88	0.39	4.80	1.60E−06	**4.15E−06**	R203K	Nucleocapsid
12	g28882	287/8	334/54	1.76	0.39	4.54	5.66E−06	**1.47E−05**	1.88	0.39	4.80	1.60E−06	**4.15E−06**	R203S**	Nucleocapsid
13	g28883	287/8	334/54	1.76	0.39	4.54	5.66E−06	**1.47E−05**	1.88	0.39	4.80	1.60E−06	**4.15E−06**	G204R	Nucleocapsid

*The analysis adjusted age and sex.

**R203S or R203R.

Three SNVs (g28881, g28882, g28883) in the nucleocapsid were in perfect linkage disequilibrium, denoted as haplotype g28881/2/3, and were found to significantly elevate the risk of hospitalization (OR = 5.81 and 6.55, q = 1.47 × 10^–5^ and 4.15 × 10^–6^ in the unadjusted and adjusted analysis, respectively). Furthermore, SNV c28854, also in nucleocapsid, was not observed among outpatients and was found to be highly associated with hospitalization (*p* = 2.03 × 10^–11^) by Fisher’s exact test.

Two SNVs (t19838 and a20268) in endoRNase were found to have significant associations with hospitalization status. The SNV t19838 mutant was absent among outpatients and was found to be significantly associated with hospitalization risk by the Fisher’s exact test (*p* = 1.09 × 10^–11^). Similarly, the SNV a20268 was found to associate with the risk of hospitalization in both unadjusted and adjusted analysis (OR = 38.47 and 42.95, q = 6.85 × 10^–4^ and 4.67 × 10^–4^, respectively). Two remaining SNVs (c1059, g25563) were found to have negative associations with COVID-19 hospitalization risk (OR = 0.46 and 0.45, *p* = 8.06 × 10^–6^ and 7.18 × 10^–6^, respectively). Similar negative associations were observed for the adjusted analysis. It is of interest to note that a single SNV a23403, coding for the well-known D614G, appeared to have no association with the COVID-19 hospitalization risk in unadjusted or adjusted analysis (*p* = 0.39 and 0.29, respectively).

### SNVs in nucleocapsid and ENDORNase have synchronized expansion patterns

By the selection threshold values, six discovered SNVs were expected to have significant and substantial expansion during the study period (Fig. 1C). The SNV haplotype g28881/2/3 started to expand prior to day 100, peaked around day 150, declined to below 10% around day 320, and re-emerged in January 2021 (black line). Following a similar temporal pattern, SNV t19839 followed a synchronized pattern with g28881/2/3, with a slightly later incline and earlier decline (red line). Similarly, two SNVs (a20268, c28854) in enodRNase and Nucleocapsid, respectively, appeared to have had a synchronized expansion pattern; proportions started to rise around day 150 and reached a plateau after day 200 (blue dash and dotted lines, respectively). Finally, SNVs c1059 and g25563, in nsp2 of ORF2ab and ORF3a, respectively, were synchronized, expanding from day 30, then contracting and expanding again towards the end of the study period (green lines). Such synchronization may imply that these variants share the same mutational histories, and thus the same haplotypes.

### SNV-haplotype (t19839-g28881-g28882-g28883) associates with COVID hospitalization risk

Focusing on four SNVs that were synchronized temporally (t19839, g28881, g28882, g28883), their haplotypic frequencies across outpatients and inpatients in the discovery case–control study were tabulated (Table 3). The reference haplotype “tggg” had frequencies of 287 (97%) and 334 (86%) copies in outpatients and inpatients respectively, while the haplotype “taac” with a single mutation was observed 8 (3%) and 11 (3%) times in outpatients and inpatients respectively. Interestingly, the haplotype “caac” was absent among outpatients completely, while it was observed 43 times (11%) among inpatients. The application of Fisher’s exact analysis suggested that this SNV haplotype was significantly associated with the COVID-19 hospitalization risk (*p*-value = 2.84 × 10^–11^).

**Table 3:** Association results of three SNV-haplotypes with hospitalization status (inpatient and outpatient) in a case–control study of 683 COVID-19 patients: frequencies of haplotypes among outpatients and inaptients, estimated coefficients, and Fisher's exact *p*-values, respectively, across three haplotypes.

Hap	Discovery Set			Replication Set			
	OUT	IN	*p*-value	OUT	IN	*p*-value	WA (%)
**H1:t19839-g28881-g28882-g28883**							
caac		43 (11.08)	2.84E−11	35 (7.35)	110 (22.54)	2.21E−10	16.83
taac	8 (2.71)	11 (2.84)		45 (9.45)	41 (8.4)		5.93
tagg				2 (0.42)	1 (0.2)		0.06
tggg	287 (97.29)	334 (86.08)		394 (82.77)	336 (68.85)		75.84
**H2:a20268-c28854**							
ac	294 (99.66)	341 (87.89)	4.56E−11	361 (75.84)	399 (81.76)	5.28E−02	87.95
at		2 (0.52)		7 (1.47)	2 (0.41)		0.60
gc	1 (0.34)	4 (1.03)		2 (0.42)	1 (0.2)		0.55
gt		40 (10.31)		106 (22.27)	86 (17.62)		8.55
nc*		1 (0.26)					1.33
**H3:c1059-g25563**							
cg	94 (31.86)	199 (51.29)	2.40E−06	195 (40.97)	239 (48.98)	7.19E−02	54.95
ct	8 (2.71)	8 (2.06)		15 (3.15)	15 (3.07)		2.70
tg	4 (1.36)	3 (0.77)		2 (0.42)	3 (0.61)		0.04
tt	189 (64.07)	176 (45.36)		264 (55.46)	231 (47.34)		41.04
yg*		2 (0.52)					0.00

Also included are haplotype frequencies in general population of Washington state (far right column).

*n—untyped nucleotide, y—ambiguity typing of either c or t.

Repeating the same haplotype tabulation in the replication case–control study yielded corresponding haplotype frequencies. Other than including a rare haplotype “tagg”, the replication analysis showed largely comparable frequencies of three SNV haplotypes, e.g., “tggg” has haplotype frequencies of 82.77% and 68.85% among outpatients and inpatients, respectively, in the replication cohort, in comparison with 97.29% and 86.08% in the discovery cohort. The haplotypic association of “tggg” with COVID-19 hospitalization risk identified in the discovery cohort was replicated in the replication cohort (*p* = 2.21 × 10^–10^).

Applying the logistic regression of hospitalization status over this SNV haplotype, haplotypic association with COVID-19 hospitalization risk with “tggg” as the reference haplotype was evaluated (Table 4). By treating “tggg” as the reference, which effectively set its coefficient to zero (OR = 1), the mutant haplotype “caac” was found to have a significantly elevated risk of COVID-19 hospitalization risk (OR = 3.69, *p* = 3.44 × 10^–10^) without adjusting any covariates. After adjusting for sex, age and a potential non-linear effect of collection time, the “caac” association was improved further (OR = 5.46, *p* = 4.71 × 10^–12^). Note that male gender and older age tended to increase risk of COVID-19 hospitalization risk from the adjusted analysis, and the squared collection time appeared to have a significant association with risk of COVID-19 hospitalization, i.e., the risk was relatively low around month of November 2020.

**Table 4:** Replication results of (t19839-g28881-g28882-g28883) with hospitalization status (inpatient and outpatient) in a case–control study of 476 outpaitents and 488 inpatients: estimated coefficients, standard error, Z-score and *p*-value across three haplotypes, from the marginal and adjusted analysis.

Hap	Unadjusted analysis					Adjusted analysis				
	Coef	OR	SE	Z	*p*	Coef	OR	SE	Z	*p*
tggg*	0.00	1.00				0.00	1.00			
caac	1.30	3.69	0.21	6.28	*3.44E−10*	1.70	5.46	0.25	6.91	*4.71E−12*
taac	0.07	1.07	0.23	0.29	7.72E−01	− 0.15	0.86	0.25	− 0.59	5.53E−01
tagg	− 0.53	0.59	1.23	− 0.44	6.63E−01	− 0.79	0.46	1.24	− 0.63	5.26E−01
Male						0.34	1.41	0.14	2.38	*1.74E−02*
Unkown sex						<< 0				
Age						0.01	1.01	0.00	3.68	*2.34E−04*
Time						− 0.01	0.99	0.00	− 1.80	7.25E−02
Time*Time						0.00	1.00	0.00	2.82	*4.76E−03*

Adjusted analysis controled sex, age, collection time and its square (to account possible non-linear time effect).

*"tggg" is treated as a reference haplotype for comparison with other haplotypes.

Because of their temporal synchrony, we next considered the haplotypic association of t20268-c28854 with hospitalization (Table 3). In the discovery set, the mutant “gt” was absent in outpatients, and was observed 10% among all inpatients. As a result, this haplotype was found to have a significant association (*p* = 4.56 × 10^–11^) in the discovery set. However, the replication analysis provided a support for this association with a marginal significance (*p* = 0.05), given 22% of outpatients carried this haplotype in comparison with 18% of inpatients. The discovered association of c1059-g25563 was replicated also with marginal significance (*p* = 0.07).

### Dynamic expansion of SNV haplotype (t19839-g28881-g28882-g28883) in Washington state

**Figure 2:**
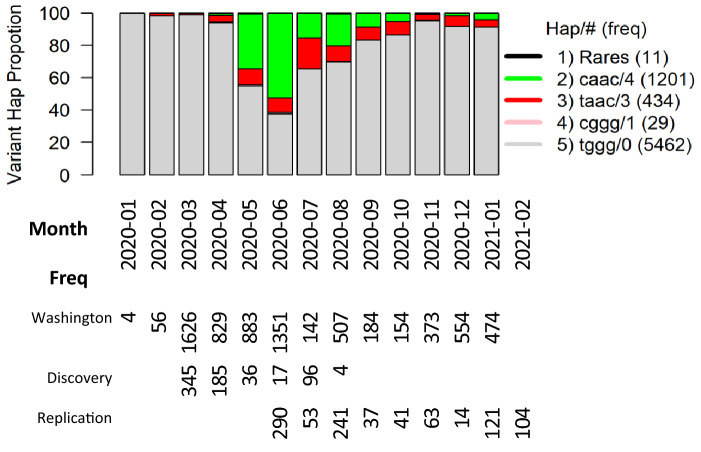
Evolving haplotype frequencies of SNV haplotype (t19839-g28881-g28882-g28883) over January 2020–2021 in Washington. Total number of samples sequenced in each month is placed below the plot. For convenience, patient numbers in discovery and replication cohorts are also included below the plot. Besides rare haplotypes, four haplotypes are annotated together with number of SNVs in each haplotype and haplotypic frequency in bracket.

The SNV haplotype t19839-g28881-g28882-g28883 has a group of seven relatively uncommon haplotypes (cagg, cgac, cggc, taaa, tagc, tag, ttgg) with fewer than 5 copies, known as rare haplotypes, and has four other relatively common haplotypes tggg/0 with 5462 copies, cggg/1 with 29 copies, taac/3 with 434 copies and caac/4 with 1201 copies, in which /# indicates the number of mutants in the haplotype. Tabulating these haplotypes over collection time by months, Fig. 2 shows that the reference haplotype tggg (gray) dominated over all months. The mutant haplotype “caac” (green), which was associated with increased risk of hospitalization, appeared in April, peaked in June, subsequently declined to a relatively low level, and then appeared to rise again in January, 2021. The other mutant haplotype taac (red) was relatively steady throughout the year, since its appearance from April, 2020. In Washington state, the reference haplotype “tggg” had a frequency of 76%, while the mutant “caac” had a frequency of 17% (Table 3).

### Classification of the haplotype (t19839c-g28881a-g28882a-g28883c)

All of the Washington viral genomes obtained from GISAID were classified by nextstrain, GISAID-clade, and lineage. To assess the relationship between the haplotype and nextstrain classification, we tabulated their cross-table frequencies (Table 5). All 1201 carriers of the “caac” haplotype were classified to 20B as were 2 carriers of “tggg”. Similarly, carriers of “caac” and “taac” belonged to the clade GR, while no carriers of the reference haplotype were assigned to that clade. Finally, with respect to the assigned lineage, 80% of “caac” carriers were assigned to lineage B.1.1.291, 7% to B.1.1.290, 6% to B.1.1, in addition to several sporadic assignments, mostly to B.1.1 (Table 6). In contrast, only 11% of the carriers of the reference haplotype “tggg” were assigned to the B.1.371 lineage.

**Table 5:** Relationships of identified SNV haplotype (t19839-g28881-g28882-g28883) with classifications of Nextstrain and clades by GISAID.

Hap	caac	taac	cggg	tggg	Rares
Nextstrain	n = 1201	434	29	5462	11
Unspecified				7 (0.13)	
19A				42 (0.77)	
19B		1 (0.23)	8 (27.59)	1422 (26.03)	1 (9.09)
20A			5 (17.24)	1032 (18.89)	4 (36.36)
20A.EU2					
20B	1201 (100)	423 (97.47)	13 (44.83)	2 (0.04)	3 (27.27)
20C			3 (10.34)	2005 (36.71)	3 (27.27)
20D		2 (0.46)			
20E (EU1)				1 (0.02)	
20F					
20G				951 (17.41)	
20H/501Y.V2					
20I/501Y.V1		8 (1.84)			
20 J/501Y.V3					
**GISAID-Clade**					
G		1 (0.23)	18 (62.07)	858 (15.71)	4 (36.36)
GH			3 (10.34)	3131 (57.32)	3 (27.27)
GR	1196 (99.58)	429 (98.85)			2 (18.18)
GV				1 (0.02)	
L					
O	5 (0.42)	2 (0.46)		35 (0.64)	1 (9.09)
S		2 (0.46)	8 (27.59)	1413 (25.87)	1 (9.09)
V				24 (0.44)

**Table 6:** Relationships of identified SNV haplotype (t19839-g28881-g28882-g28883) with lineages assigned by Pangolin.

Hap	caac	taac	cggg	tggg
Lineage	n = 1201	434*	29	5462*
A.1		2 (0.46)	8 (27.59)	1412 (25.85)
B.1		1 (0.23)	5 (17.24)	937 (17.15)
B.1.1	75 (6.24)			
B.1.1.110	1 (0.08)			
B.1.1.158	37 (3.08)		1 (3.45)	
B.1.1.222	23 (1.92)			
B.1.1.26		136 (31.34)		2 (0.04)
B.1.1.290	84 (6.99)	1 (0.23)	1 (3.45)	
B.1.1.291	956 (79.6)	7 (1.61)	11 (37.93)	
B.1.1.65		18 (4.15)		1 (0.02)
B.1.1.76	20 (1.67)			1 (0.02)
B.1.169		8 (1.84)		52 (0.95)
B.1.333.1	5 (0.42)	5 (1.15)		7 (0.13)
B.1.371			1 (3.45)	617 (11.30)
B.1.426			2 (6.90)	12 (0.22)

*Corresponding lineages associated with "taac" only or "tggg" only are excluded. For a complete list, see a separate table.

## Discussion of Washington state

This investigation utilized 7137 viral sequences obtained from Washington state from January 19, 2020 through January 31, 2021, identifying 53 SNVs that had significant expansions and maximum proportions of mutations in the last three months exceeding 10%. Through a discovery case–control design, this study discovered six SNVs associating with increased risk of COVID-19 hospitalization, while two SNVs associated with decreased hospitalization risk. Among these six SNVs, four nucleotides (c28854, g28881, g28882, g28883) were non-synonymous and code residues S194L, R203K, R203S and G204R, respectively, in Nucleocapsid, and two nucleotides (t19839, a20268) in endoRNase of orf1ab encoded synonymous changes (N73N and L216L, respectively). Interestingly, t19839 appears to have expanded together with g28881, g28882, g28883, and the combined haplotype had a significant association with COVID-19 hospitalization risk (*p* = 2.84 × 10^–11^ and 2.21 × 10^–10^ in the discovery and replication studies, respectively). Conversely, the risk association of a20268, c28854 was discovered (*p* = 4.56 × 10^–11^) but only marginally replicated (*p* = 0.05).

The non-synonymous mutations R203K, R203S and G204R in the nucleocapsid protein all occur in the flexible linker region between the N-terminal RNA-binding domain and the C-terminal dimerization domain, and this linker segment is not resolved in any reported cryo-EM or x-ray structures^4^. However, small-angle X-ray scattering (SAXS) experiments revealed that the full-length nucleocapsid protein has a much larger radius of gyration than would be expected for a 99 kDa globular protein, indicating that the flexible linker region is relatively extended in solution^4^. Consistent with the low-resolution conformational ensemble results from the SAXS experiments, recent single-molecule Förster resonance energy transfer (FRET) and fluorescence correlation spectroscopy experiments demonstrated that the linker region is highly flexible, with rapid interconversion between two general conformational populations^5^, Together, these two experimental studies show that the linker region undergoes rapid conformational transitions but is generally extended, thus minimizing direct interactions of the well-structured RNA binding and dimerization domains. The mutations R203S and G204R are non-conservative and even the R203K mutation is often observed to function as a non-conservative substitution in many cases, due to the different size of the R versus K residues and the notably different chemical features of the side-chain guanidinium group (arginine) versus the primary amine (lysine). Thus, we hypothesize that these mutations may influence disease severity by altering linker region flexibility and dynamics, which would likely alter nucleocapsid function. We also note that the linker region is involved in RNA binding interactions, so at least some of the linker region mutations might impact non-specific RNA binding.

The main variant in the endoRNase, N73N, is synonymous which suggests a testable hypotheses concerning potential impact on virus fitness or function. It is well documented that the translation kinetics for synonymous codons are often different, and this can have an impact on co-translational protein folding kinetics, yielding proteins with identical primary sequence but different conformations and properties^5^. For example, Kimchi-Sarfaty et al. demonstrated that P-glycoprotein expression using different synonymous codons yielded product with identical amino acid sequence but different substrate specificities that was attributed to differing P-glycoprotein conformations^6^. More recently, Hu et al. showed that the use of synonymous codons in heterologous expression of anti-IgE single chains in *E. coli* yielded scFv molecules with identical sequence but altered solubilities and antigen-binding affinities^7^. Thus, these synonymous mutations may lead to an endoRNase with improved function and/or properties due to alternate protein conformations. It is also possible that the synonymous mutations may impart a competitive advantage simply by resulting in enhanced translational kinetics for the endoRNase.

Many mutations in Spike protein that are correlated with increased transmission and/or severity exhibit “predictable” attributes. Specifically, such mutations are non-synonymous and occur in functionally important regions of the Spike protein where they may logically be anticipated to impact ACE2 receptor binding, alter neutralizing antibody recognition sites, or affect function via modulation of Spike protein flexibility. It is worth noting that the a23403 (D614G), the only SNV in the spike protein, was found not to associate with the severity of COVID-19 (*p* = 0.29), implying Spike protein may have a limited role in disease severity.

An interesting and important finding was that all SNVs associated with hospitalization risk were located in endoRNase or Nucleocapsid, but not in Spike protein. This observation led us to postulate that while Spike protein is essential for the transmission of the virus mediated by its binding to the angiotensin converting enzyme 2 (ACE2)^8,9^, it may play a diminished role in triggering autoimmune responses that lead to a “cytokine storm”. Instead, the presence of new mutants in Nucleocapsid may accelerate replication of the virus^10^, and endoRNase and Helicase may be responsible for initiating the secondary immune responses.

The results suggest that the viral genomes deposited in the GISAID are useful for filtering out SNVs with limited temporal patterns, allowing purpose-driven association analysis with clinical outcome data to have a sufficient power to discover phenotype-associated SNVs without sacrificing powers to correct unnecessary comparisons/tests. Furthermore, this exercise also supports a hybrid design that integrates GISAID with the purpose-driven study, given that GISAID includes sequences from the State surveillance program and are representative of the Washington study population. Direct access to electronic health records of those COVID-19 patients whose sequences have been deposited to GISAID could significantly enhance analytic rigor and findings. In essence, such a hybrid design can be viewed as a two-stage design, which has been shown to be highly efficient, and multiple statistical methodologies have been developed to extract maximum and unbiased association results^11–16^.

There are noteworthy limitations to our study including that the of hospitalization risk as a proxy for disease severity may lead to misclassification, since inpatients may be hospitalized for reasons other than COVID-19. Conversely, some patients with elevated COVID-19 hospitalization risk may not be hospitalized or their hospitalization may not be reported at the time of testing because it occurs later. However, misclassification errors tend to dilute association results^17^. Thus, the true magnitude of discovered and replicated association with the haplotype “caac” of t19839-g28881-g28882-g28883 may be even greater than estimated here if the severity of COVID-19 could be clinically adjudicated. Another limitation included incomplete matching of our discovery and replication case–control studies with regard to age, sex and collection time, partly due to challenges facing research studies relying on the operational database and biospecimen availability during the pandemic. To address this issue, we applied the logistic regression model to evaluate viral genetic associations, while adjusting for these potential confounding variables.

Successful development and implementation of COVID vaccines are expected to curtail the pandemic, but infections among unvaccinated people, and to an as yet unknown extent among the vaccinated, in and outside of the USA, is likely to generate new variants in the coming years. While ongoing genomic sequencing efforts are continuously monitoring for potential new variants, there is still a need for two-staged approach correlating viral sequencing, electronic health records, and vaccine status. Through such a strategy, viral genome sequences could be correlated with COVID-19 and vaccine related clinical outcomes, allowing for the real time identification of new variants of high consequence.

## Materials and methods

### Patient biospecimen and data

This study was approved by the Human Subject Review Committee at Fred Hutchinson Cancer Research Center (IRB#6007-2043) and by the University of Washington Institutional Review Board (STUDY00000408). The current study includes: (1) a discovery case–control study of 683 COVID-19 patients (March-August 2020), and (2) a replication case–control study of 964 patients (June 2020–March 2021) from healthcare organizations in Washington State. All subjects were de-identified, and deidentified nucleic acid samples were extracted from leftover nasal swabs and were used for viral sequencing. Deidentified demographic and healthcare-related information were extracted from electronic forms of COVID testing requests, including sex, age, and collection times (Table 1).

Treating hospitalization risk as a proxy for severity of Covid19, this study used a case–control design with inpatients as cases and outpatients as controls. In the discovery study, we attempted to match inpatients and outpatients’ sex and age by frequency as much as possible, subject to the availability of nasal swab samples. All viral sequences were deposited to Genebank (accession numbers MW593154-MW593926). The replication study included all available patients whose sequences were obtained and deposited to GISAID (https://www.gisaid.org).

This study also utilized viral genome sequences that have been deposited to GISAID (https://www.gisaid.org) from all Washington laboratories. On the downloaded dataset, we aligned all sequences against the reference genome, performing quality control, eliminated 3 samples of poor sequence quality, and removed 5’ and3’ end sequences of variable lengths. We used submission dates as a proxy for collection time, and used their classification by Nextstrain^18^, clade by GISAID (https://www.gisaid.org) and lineage by PANGO^19^.

### Samples, RNA extraction, and PCR

Patient samples were obtained and tested according to local and CDC guidelines. The University Washington (UW) Virology Division Laboratory is CLIA-certified and CAP-accredited and was one of the first academic labs in the US to offer clinical testing for SARS-CoV-2. UW Virology uses lab-developed RT-PCR tests based on either the CDC N1 and N2 or the WHO E/RdRp primer/probe sets, and FDA Emergency Use Authorization tests from Hologic (Panther Fusion), and Roche (Cobas 6800)^20–28^.

Nasopharyngeal swabs were collected in either viral transport medium (VTM) or phosphate-buffered saline (PBS). Total nucleic acid was extracted from 200 µl of VTM/PBS sample and eluted into 50 µl of buffer using MagNA Pure 96 DNA and viral NA SV Kit on MagNA Pure 96 instrument (Roche). Nucleic acids were then used for genotyping.

### Amplicon-based sequencing for discovery samples

A commercially available ScisGo®-COVID-19 kit (Scisco Genetics Inc., Seattle WA) employing an amplicon-based sequencing by synthesis approach was used to determine sequences from SARS-CoV-2 positive samples obtained from the local testing site. The approach mirrors a system previously developed for HLA and KIR typing^29,30^ using a two-stage amplicon-based PCR for locus amplification and sample barcoding and substituting two primer sets, each independently yielding non-overlapping SARS-CoV-2 amplicon sequences of ~ 400 bp. The combined derivative data spans the complete SARS-CoV-2 genome including de novo sequencing of all primer binding sites excepting the two primers at the extreme 5’ and 3’ ends. Briefly, after conversion of total nucleic acid into cDNA using the Invitrogen SuperScript IV First Strand Synthesis System (Thermo Fisher, Bothell, WA) the samples were sequentially applied to stage 1 (S1) and stage 2 (S2) PCR amplification according the manufacturer supplied protocol. After amplification, the reactions were combined, purified, and applied to a MiSeq using Illumina Version 2 chemistry with 500-cycle, paired-end sequencing (Illumina, San Diego, CA). Data assembly and analysis was performed using Sciscloud™ (Scisco Genetics Inc., Seattle WA) computational tools adapted specifically to assemble SARS-CoV-2 genomic sequences derivative from the ScisGo®-COVID-19 kit. Access to all software for data transfer and analysis was included as a component of the kit and made available through a web browser. All discovery cohort samples were sequenced using the ScisGo® approach and are accessible in genbank under accession numbers MW593154-MW593926. All other samples from UW Virology were sequenced using either metagenomic or amplicon-based approaches using the Illumina COVIDseq Test (Illumina, San Diego, CA) and the Swift Biosciences’ Normalase amplicon SARS-CoV-2 panel (Swift Biosciences, Ann Arbor, MI) as previously described^31^.

All biospecimen collections and processing were detailed in the Human Subject Research protocol, and have been reviewed and approved by the Human Subject Research Committee. With respect to statistical analyses (below), we used robust and reproducible statistical procedures that have been well-documented in the statistical literature and have been implemented in commonly accepted statistical software packages in R.

### A non-parametric logistic regression model

To model dynamic expansions and contractions of individual SNVs over time, we applied a non-parametric logistic regression model, a member of the generalized additive model (GAM), regressing a binary SNV indicators over collection times^32–34^. After fitting the model, we obtained *p*-value that quantifies the non-linear dynamics of the mutation proportion and computed the fitted values as locally weight-averaged mutation proportion daily throughout the year. The maximum proportion in the last three month, denoted as Pmax, was computed to indicate if the mutation proportion had expanded and reached a substantial level in the end of the study period. To correct multiple comparisons, the false positive error rates (q-values) were computed from *p*-values. An SNV was selected if the q-value threshold (< 0.01) indicated statistically significant dynamics and if the Pmax threshold (> 10%) suggested a substantial expansion.

### Imputing missing nucleotides

Due to the nature of sequencing technologies, a small fraction of nucleotides were untyped, and were coded as “n”. Given high linkage disequilibrium across all SNVs, we assembled a panel of polymorphic nucleotides that had no missing values and had not been selected into SNVs of interest, and treated them as an “imputation base”. Fusing one SNV with those in the imputation base, we computed their haplotype frequencies, and used their haplotype frequencies to compute posterior probabilities to impute missing nucleotides, in the same way as imputing single nucleotide polymorphisms^35^.

### Logistic regression and statistics

Treating a binary indicator of 1 and 0 for inpatient and outpatient, respectively, as an outcome, the logistic regression model regresses on SNVs or their haplotypes, to generate association statistics: estimated coefficient, standard error, Z-score, *p*-value and q-value. For SNVs with zero frequencies in either outpatients or inpatients, we performed Fisher’s exact test, instead of logistic regression model. Fisher exact test produced the exact *p*-values. Confounders (age, sex, collection time) were included into the logistic regression model as an adjusted analysis.

## Supplementary Information

Supplementary Information.

## Data Availability

All sequence data analyzed here are publicly available at GSIAD (https://www.gisaid.org/) and Genebank (https://www.ncbi.nlm.nih.gov/genbank).
