# Author Correction: Mutations in viral nucleocapsid protein and endoRNase are discovered to associate with COVID19 hospitalization risk

**DOI:** 10.1038/s41598-022-07002-z

**Published:** 2022-02-22

**Authors:** Lue Ping Zhao, Pavitra Roychoudhury, Peter Gilbert, Joshua Schiffer, Terry P. Lybrand, Thomas H. Payne, April Randhawa, Sara Thiebaud, Margaret Mills, Alex Greninger, Chul-Woo Pyo, Ruihan Wang, Renyu Li, Alexander Thomas, Brandon Norris, Wyatt C. Nelson, Keith R. Jerome, Daniel E. Geraghty

**Affiliations:** 1 Division of Public Health Sciences, Fred Hutch Cancer Center, Seattle, WA 98109 USA; 2 Vaccine and Infectious Disease Division, Fred Hutch Cancer Center, Seattle, WA 98109 USA; 3 Department of Laboratory Medicine and Pathology, University of Washington School of Medicine, Seattle, WA USA; 4 Clinical Research Division, Fred Hutch Cancer Center, Seattle, WA 98109 USA; 5 Quintepa Computing LLC, Nashville, TN USA; 6 Department of Chemistry, Department of Pharmacology, Vanderbilt University, Nashville, TN USA; 7 Department of Medicine, University of Washington School of Medicine, Seattle, WA USA; 8 Scisco Genetics Inc., Seattle, WA 98102 USA

Correction to: *Scientific Reports* 10.1038/s41598-021-04376-4, published online 24 January 2022

The original version of this Article contained an error in Figure 1, where an old version of the figure was mistakenly submitted for publication. The original Figure 1 and accompanying legend appear below.

**Figure 1:**
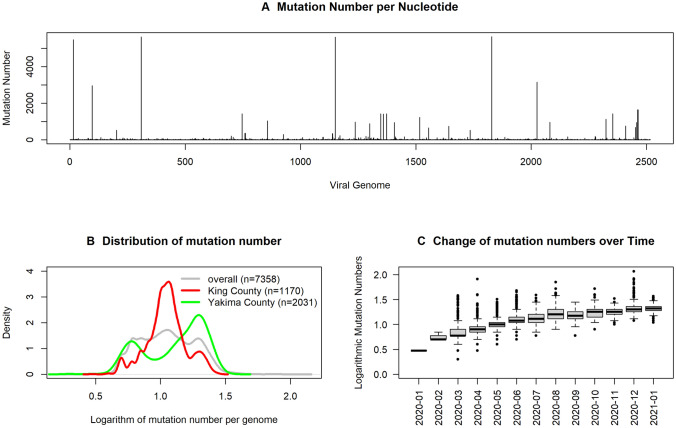
Results from analyzing 7137 viral genomes sequenced by laboratories in Washington state and deposited to GISAID. (**A**) Results from counting mutational numbers per nucleotide throughout the viral genome. Upper arrow indicates observed counts greater than 300. The viral genome is annotated with gene designations immediately below. (**B**) Computed q-values and maximum values of variant proportions in November 2020, December 2020, and January 2021, obtained from fitting generalized linear models to all individual SNVs. SNVs exceeding established threshold q-value and maximum proportions are highlighted in red (upper right corner). (**C**) Eight selected SNVs with significant and substantial temporalities are mapped using their locally averaged variant proportions over time from fitted generalized linear models (color key upper left).

The original Article has been corrected.

